# Supplementary material for: “When you’re hurt and you need serious help you call 999.” Educating children about emergency services and appropriate use of 999: An evaluation study of the Blue Light Hub app
Source: BMJ Open. 2024 Jun 10;14(6):e079214. doi: 10.1136/bmjopen-2023-079214 (PMC11168146; doi:10.1136/bmjopen-2023-079214)
Supplement: Author's manuscript [file bmjopen-2023-079214.draft_revisions.pdf]

BMJ Open is committed to open peer review. As part of this commitment we make the peer review history of every article we publish publicly available.

When an article is published we post the peer reviewers' comments and the authors' responses online. We also post the versions of the paper that were used during peer review. These are the versions that the peer review comments apply to.

The versions of the paper that follow are the versions that were submitted during the peer review process. They are not the versions of record or the final published versions. They should not be cited or distributed as the published version of this manuscript.

BMJ Open is an open access journal and the full, final, typeset and author-corrected version of record of the manuscript is available on our site with no access controls, subscription charges or pay-per-view fees (http://bmjopen.bmj.com).

If you have any questions on BMJ Open's open peer review process please email info.bmjopen@bmj.com

BMJ Open

# Educating Children about Emergency Services and Appropriate use of 999: An Evaluation Study of the Blue Light Hub App



| | |
|---|---|
| Journal: | *BMJ Open* |
| Manuscript ID | bmjopen-2023-079214 |
| Article Type: | Original research |
| Date Submitted by the Author: | 24-Aug-2023 |
| Complete List of Authors: | Paine, Amy; Cardiff University, School of Psychology<br>Maclean, Fiona; Welsh Ambulance Services NHS Trust |
| Keywords: | Health Education, ACCIDENT & EMERGENCY MEDICINE, Feasibility Studies |
| | |



SCHOLARONE™
Manuscripts

*I, the Submitting Author has the right to grant and does grant on behalf of all authors of the Work (as defined in the below author licence), an exclusive licence and/or a non-exclusive licence for contributions from authors who are: i) UK Crown employees; ii) where BMJ has agreed a CC-BY licence shall apply, and/or iii) in accordance with the terms applicable for US Federal Government officers or employees acting as part of their official duties; on a worldwide, perpetual, irrevocable, royalty-free basis to BMJ Publishing Group Ltd ("BMJ") its licensees and where the relevant Journal is co-owned by BMJ to the co-owners of the Journal, to publish the Work in this journal and any other BMJ products and to exploit all rights, as set out in our licence.*

*The Submitting Author accepts and understands that any supply made under these terms is made by BMJ to the Submitting Author unless you are acting as an employee on behalf of your employer or a postgraduate student of an affiliated institution which is paying any applicable article publishing charge ("APC") for Open Access articles. Where the Submitting Author wishes to make the Work available on an Open Access basis (and intends to pay the relevant APC), the terms of reuse of such Open Access shall be governed by a Creative Commons licence – details of these licences and which Creative Commons licence will apply to this Work are set out in our licence referred to above.*

*Other than as permitted in any relevant BMJ Author's Self Archiving Policies, I confirm this Work has not been accepted for publication elsewhere, is not being considered for publication elsewhere and does not duplicate material already published. I confirm all authors consent to publication of this Work and authorise the granting of this licence.*

For peer review only

# "When you're hurt and you need serious help you call 999"

# Educating Children about Emergency Services and Appropriate use of 999: An Evaluation Study of the Blue Light Hub App


## Abstract

**Objective.** In the face of an unprecedented demand, the Welsh Ambulance Service have developed 'Blue Light Hub': a new educational app to educate primary school-aged children about emergency services.

**Methods and Analysis.** To evaluate the effectiveness of the app, 393 primary school-aged children from three schools in South Wales played with the app for 2 hours over 2 weeks in class time. Children completed quizzes to assess their knowledge and awareness of, and confidence engaging with, emergency services before and after using the app.

**Results.** Following use of the app, there were significant increases in the proportion of children who would take appropriate action in non-emergency scenarios and knowledge of questions they could be asked if they dialled 999. There was also an increase in the proportion of children who were aware of support they could receive from the NHS, NHS job roles, and of 111.

**Conclusion.** To our knowledge, Blue Light Hub is the first app of its kind designed to educate primary school-aged children about emergency services. Our findings provide preliminary evidence that the app supports children's knowledge and awareness of emergency services.




For peer review only - http://bmjopen.bmj.com/site/about/guidelines.xhtml

The Welsh Ambulance Service handles more than 1800 calls a day from across the country. Yet of the 470,653 incidents recorded by the service in 2021, nearly a quarter were judged as non-essential and non-urgent [1]. Some reasons for non-essential engagement with emergency services may include misjudgement of emergency scenarios and perception of risk, a lack of awareness of other healthcare services, and a perception of convenience [2, 3, 4].

In the face of unprecedented demand on the service, a key priority of the Welsh Ambulance Service is to find avenues to maintain engagement with schools and influence the school curriculum to educate pupils about the Trust's services and ways to use health services appropriately [1]. Immersive and interactive experiences are an effective way to educate and familiarise children with NHS services and reduce anxieties about engaging with them, by giving children and young people opportunities to talk to NHS staff, handle equipment, and observe what might happen in different medical scenarios [5].

However, in-person interactive initiatives are resource and time-intensive and have limited opportunities for repeated exposure. Interactive apps are a useful and popular tool that is increasingly being integrated in classrooms [6]. Evidence suggests that interactive apps are a promising tool for supporting academic development [7] and for addressing public health challenges [8] such as health education [9]. By combining play with technology, interactive apps are low cost, easily distributed, and have the potential to actively engage children with material via feedback and rewards delivered through gameplay and repetition to reinforce knowledge [7, 10].

The Blue Light Hub App was designed by the Welsh Ambulance Service Trust to educate 7-to-11-year-olds about emergency services. The key objectives in the design of the interactive app were to teach children to recognise emergency from non-emergency scenarios and awareness of other services available in non-emergency situations (i.e., 111), about what happens when they call 999, the appropriate use of 999, how ambulance resources are dispatched and managed and the different uniforms they might encounter on their NHS journey. To the best of our knowledge, there is currently no other interactive app that aims to support children's engagement with emergency services. In this evaluation, we examined whether children's knowledge and awareness about, and confidence in using



For peer review only - http://bmjopen.bmj.com/site/about/guidelines.xhtml

emergency services increased following engagement with the Blue Light Hub App for two weeks in the classroom.

## Method

### Participants

Three primary schools in South Wales were invited to participate in the Blue Light Hub App evaluation. Ethical approval for the evaluation was obtained from the Cardiff University School of Psychology Ethics Committee (EC.21.12.14.6490R). For inclusivity, all children were invited to engage with the app and complete the quizzes. Consent was collected from caregivers for use of their child's data for the study. Our analyses focus on $N$ = 393 children who completed both the pre- and post-test quizzes ($n$ = 115 from School A, $n$ = 154 from School B, and $n$ = 124 from School C). On average, children were 8-9 years (median Year 4; IQR Year 3 [7-8 years] – Year 5 [9-10 years]), $n$ = 188 (47.8%) were male, $n$ = 200 (50.9%) were female (information on gender was missing for 5 participants). A G*Power post hoc power analysis for McNemar's analysis indicated that a sample size of $N$ = 393 yielded statistical power at .84 at an alpha level of .02 for proportion of discordant pairs .20, odds ratio = 2. Sociodemographic information about the schools was obtained from their most recent school inspection (Estyn) reports. At School A, approximately 37% of pupils were eligible for free school meals in their 2017 report, and at Schools B and C nearly 10% and 8% of pupils were eligible for free school meals, according to their 2016 and 2019 reports respectively (rolling three-year national average in 2021 for pupils of statutory school age who are eligible for free school meals in primary schools in Wales is 21% [11]).

### Data Collection

The Blue Light Hub App is a fully bilingual (English-Welsh) gaming app designed by Welsh Ambulance Service NHS Trust and developed by DotC Studios [12]. The app comprises four games (see Figure 1). After completing each game, children receive points that equate to money they can spend in the app's shop to buy accessories for their customisable avatar.



For peer review only

The Blue Light Hub App evaluation took place over 2 weeks in June 2022. Teachers were provided with written instructions for taking part in the evaluation. The instructions detailed that children were to be informed they could withdraw from the evaluation at any time and receive minimal support when completing their quizzes (support with reading and writing only). Children completed the first quiz in class time. After completion of the first quiz, teachers were asked to use the Blue Light Hub App with children in their class for 1 hour a week for the following 2 weeks. After these 2 weeks, children completed Quiz 2. In each quiz, children were asked questions designed to assess their knowledge about emergency scenarios, their awareness of different healthcare services and their confidence in accessing support from emergency services. Nine questions were duplicated from Quiz 1 (pre-test quiz) to Quiz 2 (post-test quiz) to enable the comparison of knowledge before and after using the Blue Light Hub App. All children received certificates from the Welsh Ambulance Service for taking part.

**Results**

Table 1 shows quiz questions of interest and children's responses. McNemar's tests were used to examine differences in proportions in children's responses from pre- to post-test quizzes; inflated alpha levels due to multiple testing were addressed with a Bonferroni adjusted significance criterion of $p < .006$. For children's knowledge about emergency services, following use of the Blue Light Hub app a higher proportion of children responded that a patient should be taken to hospital in a car if they had a minor injury (hurt arm), $\chi^2(1) = 26.01$, $p < .0005$, and provided a correct question that a call handler would ask them if they called 999, $\chi^2(1) = 13.793$, $p < .0005$. There was no significant difference in the proportion of children who reported knowing what to do if a someone had stopped breathing, $p = .18$. Children's follow-on free text responses to "If yes, what would you do?" were coded for correct actions (e.g., calling 999, administering cardiopulmonary resuscitation [CPR]). Similarly, there was no significant increase in children who said they knew how to help and gave a correct action, $p = .07$. The increase in the proportion of children who could correctly describe an emergency was also not significant, $p = .06$.



For peer review only - http://bmjopen.bmj.com/site/about/guidelines.xhtml

| | Quiz 1 (Pre-test) | Quiz 2 (Post-test) |
|---|---|---|
| Knowledge of Emergency Services | | |
| "If someone you know hurt their arm and they needed to go to hospital, what would you do?" *n* (%) take in car vs. call 999 | **70/360 (17.8)** | **126/374 (32.1)** |
| "If you saw someone and they had stopped breathing, would you know what to do?" *n* (%) yes | 306/390 (78.5) | 319/392 (81.4) |
| "If yes, what would you do?" *n* (%) correct response | 275/306 (89.9) | 299/319 (93.7) |
| "When you ring 999 for an ambulance, we ask lots of questions. Can you think of 1 question we ask you?" *n* (%) correct response | **360/393 (91.6)** | **381/393 (96.9)** |
| You should only phone 999 for an ambulance in an 'emergency'. What do you think an 'emergency' is?" *n* (%) correct response | 351/393 (89.3) | 366/393 (93.1) |
| Awareness of Healthcare Services | | |
| "If you are not feeling very well, your family and teachers can help you. Can you think of anyone else who can help you if you are not feeling very well?" *n* (%) reporting NHS service | **235/393 (58.8)** | **294/393 (74.8)** |
| "There are lots of people that do different jobs in the Ambulance Service. Can you name 2 of them?" *n* (%) correct responses | **229/393 (58.3)** | **270/393 (68.7)** |
| "Do you know how 111 can help you?" *n* (%) yes | **101/391 (25.7)** | **226/393 (57.5)** |
| Confidence Calling 999 | | |
| "Would you feel confident if you had to ring 999 for an ambulance?" *n* (%) yes | 289/389 (73.5) | 298/386 (75.8) |

*Note.* Bold represents significant differences in proportions from the pre- to post-test quizzes (McNemar)



For peer review only - http://bmjopen.bmj.com/site/about/guidelines.xhtml

In terms of children's awareness of healthcare services, we found that following use of the Blue Light Hub app, there were significant increases in the proportion of children who identified an NHS service could help them if they were not feeling well, $\chi^2(1) = 33.307$, $p < .0005$, in children who could name two roles in the NHS, $\chi^2(1) = 12.800$, $p < .0005$, and in children reporting that they knew how dialling 111 could help them, $\chi^2(1) = 90.054$, $p < .0005$ (see Table 1).

Finally, we did not identify a significant change in the proportion of children who reported feeling confident if they had to ring 999 for an ambulance $p = .31$.

## Discussion

To our knowledge, the Blue Light Hub App designed by the Welsh Ambulance Service Trust is the first of its kind to teach primary school-aged children about emergency services. Our findings suggest that using the Blue Light Hub app for just two hours over two weeks during school time was related to improvements in children's knowledge about, and awareness of, emergency services.

Importantly, following use of the app children demonstrated better understanding of when *not* to call an ambulance, suggesting the app offers opportunities to better understand actions to take in non-emergency medical scenarios. We also found that children were more likely to list NHS services that could help them if they were feeling unwell, they demonstrated a better awareness of different NHS job roles, and an increased awareness of how 111 could help them in non-emergency situations. Although we did not find significant increases in children's confidence if they were to call 999, it is noteworthy that three-quarters of children reported feeling confident and there was an increase in knowledge about what a call handler may ask them if they had to do so. Our findings extend previous research demonstrating that secondary school children tend to have high levels of knowledge about contacting emergency services [13, 14]; our evaluation indicates this is also true for younger primary school aged children. Our findings are particularly encouraging given that barriers to intervening in emergency scenarios later in childhood often involve lack of confidence, fear of making mistakes, and lack of awareness and knowledge of appropriate actions [15].

For peer review only

Although these findings are promising, there are limitations to note. This evaluation represents a first step in understanding the feasibility and effectiveness of an educational app to support children's knowledge of emergency services, however, future evaluation of the effectiveness of app as an intervention for children should be investigated in the context of a randomized controlled trial to evaluate the effectiveness of the app in the short- and long-term, that also accounts for the level of support that teachers provide when navigating the app. Given the short-term use of the app in this evaluation, a further avenue for investigation will be the long-term retention of knowledge and the potential need for repeated use of the app in class time. Additionally, future research would do well to understand how the app can be used to consolidate children's knowledge of emergency services alongside in-person interactive initiatives.

In summary, our findings suggest that primary school aged children's knowledge and awareness of emergency scenarios and services can be supported via educational apps, such as the Blue Light Hub app. Our findings corroborate research demonstrating the value of educating children in what to do in emergency scenarios and emergency care [5], and that primary school-aged children are receptive to interventions that support their ability to recognise emergencies and call for help [16, 17]. We join other calls for more emphasis on emergency response readiness in school curriculums [15, 18, 19]; this may have positive consequences for wider community knowledge of appropriate use of emergency services. Our findings, though preliminary, suggest that amongst other educational approaches, educational apps may be a useful resource for teaching.

## Acknowledgments

We would like to thank the children and teachers who took part in this study. Dr. Amy L. Paine is funded by an ESRC New Investigator Award (Grant reference: ES/T00049X/1). We would also like to thank Cara Houlcroft, Meg Kydd-Coutts, Maisie Bowmaster, and Anna Preece for their research assistance. This research was conducted in consultation with the Welsh Ambulance Service NHS Trust (WAST), but WAST did not have final say in the study design nor played a direct role in the collection or analysis of the data; interpretation of the results; or writing of the manuscript. PPI



For peer review only - http://bmjopen.bmj.com/site/about/guidelines.xhtml

Statement: Patients or the public were not involved in the design, or conduct, or reporting, or dissemination plans of our research.

## References


1. Welsh Ambulance Service NHS Trust. '999? I've eaten a mouldy tomato!' – Inappropriate calls to Welsh Ambulance Service revealed [online]. 2022. https://www.ambulance.wales.nhs.uk/Default.aspx?gcid=1862&lan=en#:~:text=THE%20Welsh%20Ambulance%20Service%20has%20revealed%20the%20most,Department%2C%20while%20another%20dialled%20999%20for%20a%20papercut (accessed 21 March 2023).

2. Ahl C, Nyström M, Jansson L. Making up one's mind: – Patients' experiences of calling an ambulance. Accident and Emergency Nursing 2006; 14: 11-19. doi: 10.1016/j.aaen.2005.10.002

3. Brooker MJ, Shaw ARG, Purdy S. Why do patients with 'primary care sensitive' problems access ambulance services? A systematic mapping review of the literature. BMJ Open 2015; 5: e007726. doi: 10.1136/bmjopen-2015-007726

4. Chapman B, Turnbull T. Health professionals' perceptions of inappropriate use of A&E services. British Journal of Nursing 2016; 25. doi: 10.12968/bjon.2016.25.9.476

5. Allen D. 999 Club aims to reduce children's anxieties about emergency care. Emergency Nurse 2018; 25: 8. doi: 10.7748/en.25.10.8.s8

6. Haßler B, Major L, Hennessy S. Tablet use in schools: a critical review of the evidence for learning outcomes. Journal of Computer Assisted Learning 2016; 32: 139–156. doi: 10.1111/jcal.12123

7. Griffith SF et al. Apps As Learning Tools: A Systematic Review. Pediatrics 2020; 145: e20191579. 10.1542/peds.2019-1579

8. World Health Organization. WHO Guideline: Recommendations on Digital Interventions for Health System Strengthening [online]. 2019.




For peer review only - http://bmjopen.bmj.com/site/about/guidelines.xhtml

https://apps.who.int/iris/bitstream/handle/10665/324998/WHO-RHR-19.7-eng.pdf (accessed 21 March 2023).

9. Brown JM et al. Optimizing Child Nutrition Education with the Foodbot Factory Mobile Health App: Formative Evaluation and Analysis. JMIR Formative Research 2020; 4:e15534. doi: 10.2196/15534

10. Hirsh-Pasek K et al. Putting education in "educational" apps: lessons from the science of learning. Psychological Science in the Public Interest, 2015; 16: 3–34. doi: 10.1177/1529100615569721

11. Estyn. The teaching of Welsh history including Black, Asian and Minority Ethnic history, identity and culture [online] 2021. https://www.estyn.gov.wales/document-page/20673/appendices/appendix-3#:~:text=The%20rolling%20three-year%20national%20average%20in%202021%20for,Wales%20have%20a%20special%20educational%20needs%20provision%20%28SEN%29 (accessed 21 March 2023).

12. DotC Studios [online]. 2023. https://dotcstudios.com/ (accessed 21 March 2023).

13. Kanstad B K et al. CPR knowledge and attitude to performing bystander CPR among secondary school students in Norway. Resuscitation, 2011, 82: 1053-1059. doi:10.1016/j.resuscitation. 2011.03.033

14. Schuffelen P. Knowledge of the European emergency number in secondary schools for different grades and education level groups. Resuscitation, 2013; 84(Suppl 1): S40. doi: 10.1016/j.resuscitation.2013.08.105

15. Parnell M M et al. Knowledge of and attitudes towards resuscitation in New Zealand high-school students. Emergency Medicine Journal, 2006, 23: 899-902. 10.1136/emj.2006.041160

16. Bernardo L M et al. Basic Emergency Lifesaving Skills (BELS): A framework for teaching skills to children and adolescents. International Journal of Trauma Nursing, 2002, 8: 48-50. doi: 10.1067/mtn.2002.123027



For peer review only - http://bmjopen.bmj.com/site/about/guidelines.xhtml

17. Wilks J et al. Emergency response readiness for primary school children. Australian Health Review, 2015; 40: 357-363. doi: 10.1071/AH15072

18. Bollig G et al. Primary school children are able to perform basic life-saving first aid measures. Resuscitation, 2009; 80: 689-692. doi: 10.1016/j.resuscitation.2009.03.012

19. Uray T et al. Feasibility of life-supporting first-aid (LSFA) training as a mandatory subject in primary schools. Resuscitation, 2003; 59: 211-220. doi: 10.1016/S0300-9572(03)00233-8

For peer review only



For peer review only - http://bmjopen.bmj.com/site/about/guidelines.xhtml

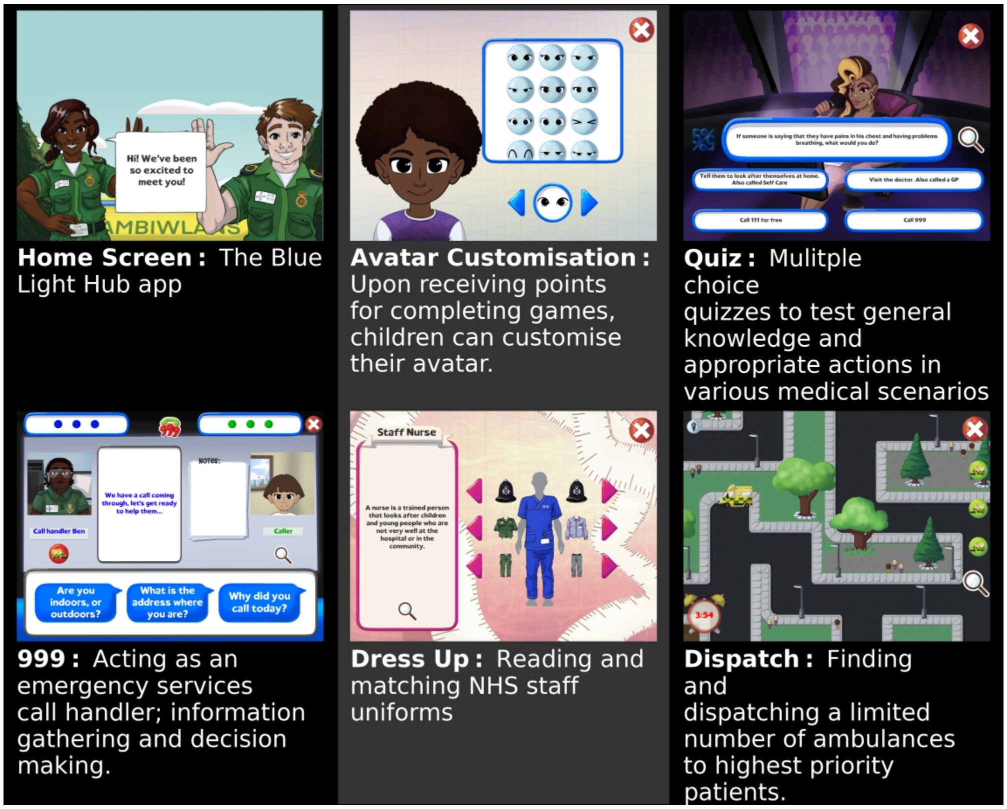


Figure 1. The Blue Light Hub App

149x119mm (600 x 600 DPI)

BMJ Open

# “When you're hurt and you need serious help you call 999” Educating Children about Emergency Services and Appropriate use of 999: An Evaluation Study of the Blue Light Hub App



| | |
|---|---|
| Journal: | *BMJ Open* |
| Manuscript ID | bmjopen-2023-079214.R1 |
| Article Type: | Original research |
| Date Submitted by the Author: | 23-Feb-2024 |



| | |
|---|---|
| Complete List of Authors: | Paine, Amy; Cardiff University, School of Psychology<br>Maclean, Fiona; Welsh Ambulance Services NHS Trust |



| | |
|---|---|
| <b>Primary Subject Heading</b>: | Medical education and training |
| Secondary Subject Heading: | Emergency medicine, Communication, Public health |
| Keywords: | Health Education, ACCIDENT & EMERGENCY MEDICINE, Feasibility Studies |
| | |


SCHOLARONE™
Manuscripts

*I, the Submitting Author has the right to grant and does grant on behalf of all authors of the Work (as defined in the below author licence), an exclusive licence and/or a non-exclusive licence for contributions from authors who are: i) UK Crown employees; ii) where BMJ has agreed a CC-BY licence shall apply, and/or iii) in accordance with the terms applicable for US Federal Government officers or employees acting as part of their official duties; on a worldwide, perpetual, irrevocable, royalty-free basis to BMJ Publishing Group Ltd ("BMJ") its licensees and where the relevant Journal is co-owned by BMJ to the co-owners of the Journal, to publish the Work in this journal and any other BMJ products and to exploit all rights, as set out in our licence.*

*The Submitting Author accepts and understands that any supply made under these terms is made by BMJ to the Submitting Author unless you are acting as an employee on behalf of your employer or a postgraduate student of an affiliated institution which is paying any applicable article publishing charge ("APC") for Open Access articles. Where the Submitting Author wishes to make the Work available on an Open Access basis (and intends to pay the relevant APC), the terms of reuse of such Open Access shall be governed by a Creative Commons licence – details of these licences and which Creative Commons licence will apply to this Work are set out in our licence referred to above.*

*Other than as permitted in any relevant BMJ Author's Self Archiving Policies, I confirm this Work has not been accepted for publication elsewhere, is not being considered for publication elsewhere and does not duplicate material already published. I confirm all authors consent to publication of this Work and authorise the granting of this licence.*

For peer review only

# "When you're hurt and you need serious help you call 999"

# Educating Children about Emergency Services and Appropriate use of 999: An Evaluation Study of the Blue Light Hub App


## Abstract

**Objective.** In the face of an unprecedented demand, the Welsh Ambulance Service have developed 'Blue Light Hub': a new educational app to educate primary school-aged children about emergency services.

**Methods and Analysis.** To evaluate the effectiveness of the app, 393 primary school-aged children from three schools in South Wales played with the app for 2 hours over 2 weeks in class time. Children completed quizzes to assess their knowledge and awareness of, and confidence engaging with, emergency services before and after using the app.

**Results.** Following use of the app, there were significant increases in the proportion of children who would take appropriate action in non-emergency scenarios and knowledge of questions they could be asked if they dialled 999. There was also an increase in the proportion of children who were aware of support they could receive from the NHS, NHS job roles, and of 111.

**Conclusion.** To our knowledge, Blue Light Hub is the first app of its kind designed to educate primary school-aged children about emergency services. Our findings provide preliminary evidence that the app supports children's knowledge and awareness of emergency services.




For peer review only - http://bmjopen.bmj.com/site/about/guidelines.xhtml

**Strengths and limitations of this study**

- To our knowledge, this study was the first to evaluate the effectiveness of an educational app designed to support children's engagement with emergency services.
- The Blue Light Hub app offers a low-cost and easy-to-disseminate method of improving children's knowledge and awareness of emergency services.
- Future studies should investigate effectiveness of the app in the long-term and assess generalisability across different contexts.
- Further development of the app, or similar apps, should harness the views of children, parents, and teachers via advisory groups alongside the lessons learned from this initial evaluation.

For peer review only

The Welsh Ambulance Service handles more than 1800 calls a day from across the country. Yet of the 470,653 incidents recorded by the service in 2021, nearly a quarter were judged as non-essential and non-urgent [1]. Some reasons for non-essential engagement with emergency services may include misjudgement of emergency scenarios and perception of risk, a lack of awareness of other healthcare services, and a perception of convenience [2, 3, 4].

In the face of unprecedented demand on the service, a key priority of the Welsh Ambulance Service is to find avenues to maintain engagement with schools and influence the school curriculum to educate pupils about the Trust's services and ways to use health services appropriately [1]. Immersive and interactive experiences are an effective way to educate and familiarise children with NHS services and reduce anxieties about engaging with them, by giving children and young people opportunities to talk to NHS staff, handle equipment, and observe what might happen in different medical scenarios [5].

However, in-person interactive initiatives are resource and time-intensive and have limited opportunities for repeated exposure. Interactive apps are a useful and popular tool that is increasingly being integrated in classrooms [6]. Evidence suggests that interactive apps are a promising tool for supporting academic development [7] and for addressing public health challenges [8] such as health education [9]. By combining play with technology, interactive apps are low cost, easily distributed, and have the potential to actively engage children with material via feedback and rewards delivered through gameplay and repetition to reinforce knowledge [7, 10].

The Blue Light Hub App was designed by the Welsh Ambulance Service Trust to educate 7-to-11-year-olds about emergency services. The key objectives in the design of the interactive app were to teach children to recognise emergency from non-emergency scenarios and awareness of other services available in non-emergency situations (i.e., 111), about what happens when they call 999, the appropriate use of 999, how ambulance resources are dispatched and managed and the different uniforms they might encounter on their NHS journey. To the best of our knowledge, there is currently no other interactive app that aims to support children's engagement with emergency services. In this evaluation, we examined the following questions: Does the use of the Blue Light Hub app increase



For peer review only - http://bmjopen.bmj.com/site/about/guidelines.xhtml

children's children's (1) knowledge of (2) awareness about, and (3) confidence in using emergency services? These questions were investigated in the context of the Blue Light Hub App being used by primary school-aged children for two weeks in the classroom.

## Method

### Participants

Three primary schools in South Wales were invited to participate in the Blue Light Hub App evaluation. Ethical approval for the evaluation was obtained from the Cardiff University School of Psychology Ethics Committee (EC.21.12.14.6490R). For inclusivity, all children were invited to engage with the app and complete the quizzes. Consent was collected from caregivers for use of their child's data for the study. Our analyses focus on $N = 393$ children who completed both the pre- and post-test quizzes ($n = 115$ from School A, $n = 154$ from School B, and $n = 124$ from School C). On average, children were 8-9 years (median Year 4; IQR Year 3 [7-8 years] – Year 5 [9-10 years]), $n = 188$ (47.8%) were male, $n = 200$ (50.9%) were female (information on gender was missing for 5 participants). Sociodemographic information about the schools was obtained from their most recent school inspection (Estyn) reports. At School A, approximately 37% of pupils were eligible for free school meals in their 2017 report, and at Schools B and C nearly 10% and 8% of pupils were eligible for free school meals, according to their 2016 and 2019 reports respectively (rolling three-year national average in 2021 for pupils of statutory school age who are eligible for free school meals in primary schools in Wales is 21% [11]).

### Data Collection

The Blue Light Hub App is a fully bilingual (English-Welsh) gaming app designed by Welsh Ambulance Service NHS Trust and developed by DotC Studios [12]. The app comprises four games (see Figure 1). After completing each game, children receive points that equate to money they can spend in the app's shop to buy accessories for their customisable avatar.

The Blue Light Hub App evaluation took place over 2 weeks in June 2022. Teachers were provided with written instructions for taking part in the evaluation. The instructions detailed that



For peer review only - http://bmjopen.bmj.com/site/about/guidelines.xhtml

children were to be informed they could withdraw from the evaluation at any time and receive minimal support when completing their quizzes (support with reading and writing only). Children completed the first quiz in class time. After completion of the first quiz, teachers were asked to use the Blue Light Hub App with children in their class for 1 hour a week for the following 2 weeks. After these 2 weeks, children completed Quiz 2. In each quiz, children were asked questions designed to assess their knowledge about emergency scenarios, their awareness of different healthcare services and their confidence in accessing support from emergency services. Nine questions were duplicated from Quiz 1 (pre-test quiz) to Quiz 2 (post-test quiz) to enable the comparison of knowledge before and after using the Blue Light Hub App. All children received certificates from the Welsh Ambulance Service for taking part.

**Data Analysis.**

Quiz responses were analysed using SPSS software V.27 (IBM). Change in knowledge, awareness, and confidence before and after use of the Blue Light Hub app was assessed using McNemar's tests, to examine differences in proportions in children's responses from pre- to post-test quizzes. Inflated alpha levels due to multiple testing were addressed with a Bonferroni adjusted significance criterion of $p < .006$. A G*Power post hoc power analysis for McNemar's analysis indicated that a sample size of $N = 393$ yielded statistical power at .84 at an alpha level of .02 for proportion of discordant pairs .20, odds ratio = 2.

## Results

Table 1 shows quiz questions of interest and children's responses. For children's knowledge about emergency services, following use of the Blue Light Hub app a higher proportion of children responded that a patient should be taken to hospital in a car if they had a minor injury (hurt arm), $\chi^2(1) = 26.01$, $p < .0005$, $OR = 4.38$, 95% $CI$(2.47 to 7.77), and provided a correct question that a call handler would ask them if they called 999, $\chi^2(1) = 13.793$, $p < .0005$ ($OR = 28.48$, 95% $CI$(8.02 to 101.10); but it is worthy of note that a very high proportion of children correctly answered this question at both time points. There was no significant difference in the proportion of children who



For peer review only - http://bmjopen.bmj.com/site/about/guidelines.xhtml

reported knowing what to do if a someone had stopped breathing, $p = .18$. Children's follow-on free text responses to "If yes, what would you do?" were coded for correct actions (e.g., calling 999, administering cardiopulmonary resuscitation [CPR]). Similarly, there was no significant increase in children who said they knew how to help and gave a correct action, $p = .07$. The increase in the proportion of children who could correctly describe an emergency was also not significant, $p = .06$.

**Table 1. Children's quiz results before and after playing with the Blue Light Hub app**

| | Quiz 1 (Pre-test) | Quiz 2 (Post-test) |
|---|---|---|
| Knowledge of Emergency Services | | |
| "If someone you know hurt their arm and they needed to go to hospital, what would you do?" *n* (%) take in car vs. call 999 | **70/360 (17.8)** | **126/374 (32.1)** |
| "If you saw someone and they had stopped breathing, would you know what to do?" *n* (%) yes | 306/390 (78.5) | 319/392 (81.4) |
| "If yes, what would you do?" *n* (%) correct response | 275/306 (89.9) | 299/319 (93.7) |
| "When you ring 999 for an ambulance, we ask lots of questions. Can you think of 1 question we ask you?" *n* (%) correct response | **360/393 (91.6)** | **381/393 (96.9)** |
| You should only phone 999 for an ambulance in an 'emergency'. What do you think an 'emergency' is?" *n* (%) correct response | 351/393 (89.3) | 366/393 (93.1) |
| Awareness of Healthcare Services | | |
| "If you are not feeling very well, your family and teachers can help you. Can you think of anyone else who can help you if you are not feeling very well?" *n* (%) reporting NHS service | **235/393 (58.8)** | **294/393 (74.8)** |
| "There are lots of people that do different jobs in the Ambulance Service. Can you name 2 of them?" *n* (%) correct responses | **229/393 (58.3)** | **270/393 (68.7)** |



For peer review only - http://bmjopen.bmj.com/site/about/guidelines.xhtml

| | | |
|---|---|---|
| "Do you know how 111 can help you?" *n* (%) yes | **101/391 (25.7)** | **226/393 (57.5)** |
| Confidence Calling 999 | | |
| "Would you feel confident if you had to ring 999 for an ambulance?" *n* (%) yes | 289/389 (73.5) | 298/386 (75.8) |

*Note.* Bold represents significant differences in proportions from the pre- to post-test quizzes.

In terms of children's awareness of healthcare services, we found that following use of the Blue Light Hub app, there were significant increases in the proportion of children who identified an NHS service could help them if they were not feeling well, $\chi^2(1) = 33.307$, $p < .0005$, $OR = 9.94$, 95% $CI$(5.76 to 17.15), in children who could name two roles in the NHS, $\chi^2(1) = 12.800$, $p < .0005$, $OR = 4.35$, 95% $CI$(2.76 to 6.84), and in children reporting that they knew how dialling 111 could help them, $\chi^2(1) = 90.054$, $p < .0005$, $OR = 3.54$, 95% $CI$(2.09 to 5.99), (see Table 1).

Finally, we did not identify a significant change in the proportion of children who reported feeling confident if they had to ring 999 for an ambulance $p = .31$.

## Discussion

To our knowledge, the Blue Light Hub App designed by the Welsh Ambulance Service Trust is the first of its kind to teach primary school-aged children about emergency services. Our findings suggest that using the Blue Light Hub app for just two hours over two weeks during school time was related to improvements in children's knowledge about, and awareness of, emergency services.

Importantly, following use of the app children demonstrated better understanding of when *not* to call an ambulance, suggesting the app offers opportunities to better understand actions to take in non-emergency medical scenarios. We also found that children were more likely to list NHS services that could help them if they were feeling unwell, they demonstrated a better awareness of different NHS job roles, and an increased awareness of how 111 could help them in non-emergency situations. Although we did not find significant increases in children's confidence if they were to call 999, it is



For peer review only - http://bmjopen.bmj.com/site/about/guidelines.xhtml
For peer review only

noteworthy that three-quarters of children reported feeling confident and there was an increase in knowledge about what a call handler may ask them if they had to do so. Our findings extend previous research demonstrating that secondary school children tend to have high levels of knowledge about contacting emergency services [13, 14]; our evaluation indicates this is also true for younger primary school aged children. Our findings are particularly encouraging given that barriers to intervening in emergency scenarios later in childhood often involve lack of confidence, fear of making mistakes, and lack of awareness and knowledge of appropriate actions [15].

One of the main aims of developing the Blue Light Hub app was to find novel ways to support education about urgent and emergency care to address the rise in calls for ambulance services in non-emergency scenarios. Such calls add significant pressure to an already stretched service that needs to reduce spending [16]. Research shows that reasons for ambulance use in non-emergency situations are multifaceted [2, 3, 4]. For example, recent reviews have indicated numerous factors associated with ambulance use for non-emergency problems. These included socioeconomic status, geographical location, practical reasons (i.e., lack of transport), emotional reasons (i.e., uncertainty, reassurance), and level of education, knowledge, and experience in judging the appropriateness of ambulance use for their circumstances [17, 18]. Although a small-scale intervention may not radically change the behaviour of the population, better knowledge and awareness repeatedly consolidated by use of educational apps may encourage individuals to evaluate their options more thoroughly before seeking emergency care. Quite possibly, interventions such as the Blue Light Hub app may have most impact in more deprived areas; future research would do well to investigate this possibility on a larger scale and explore the generalisability of this type of education-based intervention in other locations and contexts. Although this investigation focused on improving awareness, knowledge, and confidence in primary school aged children, app-based education could also be explored as an avenue to supporting adult education about emergency services and decision making prior to calling an ambulance.

For peer review only

**Improvements**

The app was developed during the Covid-19 pandemic, and therefore opportunities for consultation with young people, parents, and teachers were limited. We recommend that future development of such apps involve the inclusion of both a young person and parent and teacher advisory groups alongside the lessons learned from this this initial evaluation.

In general feedback, children and teachers shared that the Blue Light Hub app was engaging and enjoyable to use. However, this initial evaluation gave insights into ways the app can be improved in terms of accessibility and improving children's learning experiences. Some teachers suggested that the app should include audio to reduce the need for reading skills and increase accessibility of the app. Another popular suggestion by teachers was further consideration that children typically share tablet devices in the classroom and across year groups. An important step for further development of the app would be to ensure that children can access their own profiles to be able to keep track of their own progress and learning journey.

Children had many ideas for more games they would want to play on the app, including how to give CPR, first aid, and more immersive experiences of being an ambulance or air ambulance driver. Indeed, one valuable direction for game development could be an experience of steps to take if a child encountered a patient who required CPR, given that evidence suggests that in later childhood, despite good knowledge of actions to take, this does not necessarily translate to practical ability [19].

**Limitations**

Although these findings are promising, there are limitations to note. This evaluation represents a first step in understanding the feasibility and effectiveness of an educational app to support children's knowledge of emergency services, however, future evaluation of the effectiveness of app as an intervention for children should be investigated in the context of a randomized controlled trial to evaluate the effectiveness of the app in the short- and long-term, that also accounts for the level of support that teachers provide when navigating the app. Given the short-term use of the app in this evaluation, a further avenue for investigation will be the long-term retention of knowledge and



For peer review only - http://bmjopen.bmj.com/site/about/guidelines.xhtml

the potential need for repeated use of the app in class time. Additionally, future research would do well to understand how the app can be used to consolidate children's knowledge of emergency services alongside in-person interactive initiatives.

**Conclusion**

In summary, our findings suggest that primary school aged children's knowledge and awareness of emergency scenarios and services can be supported via educational apps, such as the Blue Light Hub app. Our findings corroborate research demonstrating the value of educating children in what to do in emergency scenarios and emergency care [5], and that primary school-aged children are receptive to interventions that support their ability to recognise emergencies and call for help [20, 21]. We join other calls for more emphasis on emergency response readiness in school curriculums [15, 22, 23]; this may have positive consequences for wider community knowledge of appropriate use of emergency services. Our findings, though preliminary, suggest that amongst other educational approaches, educational apps may be a useful resource for teaching.

**Contributorship statement.** Author AP designed the study, monitored data collection, planned the analyses, drafted, and revised the paper. Author FM led recruitment, monitored data collection, and edited the paper. We would also like to thank Cara Houlcroft, Meg Kydd-Coutts, Maisie Bowmaster, and Anna Preece for their research assistance. We would like to thank the children and teachers who took part in this study. PPI Statement: Patients or the public were not involved in the design, or conduct, or reporting, or dissemination plans of our research.

**Competing interests:** This research was conducted in consultation with the Welsh Ambulance Service NHS Trust (WAST), but WAST did not have final say in the study design nor played a direct role in the collection or analysis of the data; interpretation of the results; or writing of the manuscript.

**Funding:** Dr. Amy L. Paine is funded by an ESRC New Investigator Award (Grant reference: ES/T00049X/1).

**Data sharing statement:** Data are available from the research team upon reasonable request.



For peer review only - http://bmjopen.bmj.com/site/about/guidelines.xhtml

## References


1. Welsh Ambulance Service NHS Trust. '999? I've eaten a mouldy tomato!' – Inappropriate calls to Welsh Ambulance Service revealed [online]. 2022. https://www.ambulance.wales.nhs.uk/Default.aspx?gcid=1862&lan=en#:~:text=THE%20Welsh%20Ambulance%20Service%20has%20revealed%20the%20most,Department%2C%20while%20another%20dialled%20999%20for%20a%20papercut (accessed 21 March 2023).

2. Ahl C, Nyström M, Jansson L. Making up one's mind: – Patients' experiences of calling an ambulance. Accident and Emergency Nursing 2006; 14: 11-19. doi: 10.1016/j.aaen.2005.10.002

3. Brooker MJ, Shaw ARG, Purdy S. Why do patients with 'primary care sensitive' problems access ambulance services? A systematic mapping review of the literature. BMJ Open 2015; 5: e007726. doi: 10.1136/bmjopen-2015-007726

4. Chapman B, Turnbull T. Health professionals' perceptions of inappropriate use of A&E services. British Journal of Nursing 2016; 25. doi: 10.12968/bjon.2016.25.9.476

5. Allen D. 999 Club aims to reduce children's anxieties about emergency care. Emergency Nurse 2018; 25: 8. doi: 10.7748/en.25.10.8.s8

6. Haßler B, Major L, Hennessy S. Tablet use in schools: a critical review of the evidence for learning outcomes. Journal of Computer Assisted Learning 2016; 32: 139–156. doi: 10.1111/jcal.12123

7. Griffith SF et al. Apps As Learning Tools: A Systematic Review. Pediatrics 2020; 145: e20191579. 10.1542/peds.2019-1579

8. World Health Organization. WHO Guideline: Recommendations on Digital Interventions for Health System Strengthening [online]. 2019. https://apps.who.int/iris/bitstream/handle/10665/324998/WHO-RHR-19.7-eng.pdf (accessed 21 March 2023).




For peer review only - http://bmjopen.bmj.com/site/about/guidelines.xhtml

9. Brown JM et al. Optimizing Child Nutrition Education with the Foodbot Factory Mobile Health App: Formative Evaluation and Analysis. JMIR Formative Research 2020; 4:e15534. doi: 10.2196/15534

10. Hirsh-Pasek K et al. Putting education in "educational" apps: lessons from the science of learning. Psychological Science in the Public Interest, 2015; 16: 3–34. doi: 10.1177/1529100615569721

11. Estyn. The teaching of Welsh history including Black, Asian and Minority Ethnic history, identity and culture [online] 2021. https://www.estyn.gov.wales/document-page/20673/appendices/appendix-3#:~:text=The%20rolling%20three-year%20national%20average%20in%202021%20for,Wales%20have%20a%20special%20educational%20needs%20provision%20%28SEN%29 (accessed 21 March 2023).

12. DotC Studios [online]. 2023. https://dotcstudios.com/ (accessed 21 March 2023).

13. Kanstad B K et al. CPR knowledge and attitude to performing bystander CPR among secondary school students in Norway. Resuscitation, 2011, 82: 1053-1059. doi: 10.1016/j.resuscitation.2011.03.033

14. Schuffelen P. Knowledge of the European emergency number in secondary schools for different grades and education level groups. Resuscitation, 2013; 84(Suppl 1): S40. doi: 10.1016/j.resuscitation.2013.08.105

15. Parnell M M et al. Knowledge of and attitudes towards resuscitation in New Zealand high-school students. Emergency Medicine Journal, 2006, 23: 899-902. doi: 10.1136/emj.2006.041160

16. NHS England. High quality care for all, now and for future generations: Transforming urgent and emergency care services in England. The evidence base from the urgent and primary care review [online] 2013. https://www.england.nhs.uk/wp-content/uploads/2013/06/urg-emerg-care-ev-bse.pdf

17. Proctor A et al. What factors are associated with ambulance use for non-emergency problems in children? A systematic mapping review and qualitative synthesis. BMJ Open, 2021, 11(9): e049443. doi: 10.1136/bmjopen-2021-049443

For peer review only

18. Booker M J et al. Seeking ambulance treatment for ‘primary care’ problems: a qualitative systematic review of patient, carer and professional perspectives. BMJ Open, 2017, 7(8): e016832. doi: 10.1136/bmjopen-2017-016832

19. Lester C et al. Teaching schoolchildren cardiopulmonary resuscitation. Resuscitation, 1996, 31(1): 33-88. doi: 10.1016/0300-9572(95)00912-4

20. Bernardo L M et al. Basic Emergency Lifesaving Skills (BELS): A framework for teaching skills to children and adolescents. International Journal of Trauma Nursing, 2002, 8: 48-50. doi: 10.1067/mtn.2002.123027

21. Wilks J et al. Emergency response readiness for primary school children. Australian Health Review, 2015; 40: 357-363. doi: 10.1071/AH15072

22. Bollig G et al. Primary school children are able to perform basic life-saving first aid measures. Resuscitation, 2009; 80: 689-692. doi: 10.1016/j.resuscitation.2009.03.012

23. Uray T et al. Feasibility of life-supporting first-aid (LSFA) training as a mandatory subject in primary schools. Resuscitation, 2003; 59: 211-220. doi: 10.1016/S0300-9572(03)00233-8

For peer review only



For peer review only - http://bmjopen.bmj.com/site/about/guidelines.xhtml

Figure caption:

**Figure 1. The Blue Light Hub App**

For peer review only

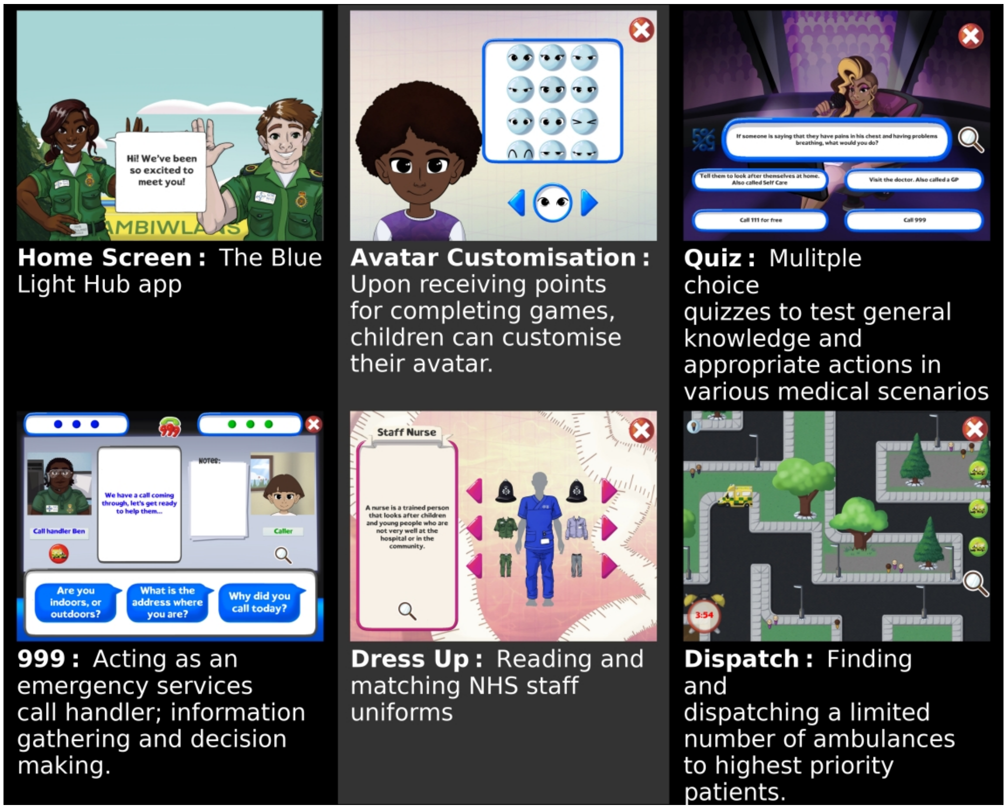


Figure 1. The Blue Light Hub App

149x119mm (600 x 600 DPI)

BMJ Open

# “When you're hurt and you need serious help you call 999” Educating Children about Emergency Services and Appropriate use of 999: An Evaluation Study of the Blue Light Hub App


| | |
|---|---|
| Journal: | *BMJ Open* |
| Manuscript ID | bmjopen-2023-079214.R2 |
| Article Type: | Original research |
| Date Submitted by the Author: | 03-May-2024 |
| Complete List of Authors: | Paine, Amy; Cardiff University, School of Psychology<br>Maclean, Fiona; Welsh Ambulance Services NHS Trust |
| <b>Primary Subject Heading</b>: | Medical education and training |
| Secondary Subject Heading: | Emergency medicine, Communication, Public health |
| Keywords: | Health Education, ACCIDENT & EMERGENCY MEDICINE, Feasibility Studies |
| | |


SCHOLARONE™
Manuscripts

For peer review only - http://bmjopen.bmj.com/site/about/guidelines.xhtml

*I, the Submitting Author has the right to grant and does grant on behalf of all authors of the Work (as defined in the below author licence), an exclusive licence and/or a non-exclusive licence for contributions from authors who are: i) UK Crown employees; ii) where BMJ has agreed a CC-BY licence shall apply, and/or iii) in accordance with the terms applicable for US Federal Government officers or employees acting as part of their official duties; on a worldwide, perpetual, irrevocable, royalty-free basis to BMJ Publishing Group Ltd ("BMJ") its licensees and where the relevant Journal is co-owned by BMJ to the co-owners of the Journal, to publish the Work in this journal and any other BMJ products and to exploit all rights, as set out in our licence.*

*The Submitting Author accepts and understands that any supply made under these terms is made by BMJ to the Submitting Author unless you are acting as an employee on behalf of your employer or a postgraduate student of an affiliated institution which is paying any applicable article publishing charge ("APC") for Open Access articles. Where the Submitting Author wishes to make the Work available on an Open Access basis (and intends to pay the relevant APC), the terms of reuse of such Open Access shall be governed by a Creative Commons licence – details of these licences and which Creative Commons licence will apply to this Work are set out in our licence referred to above.*

*Other than as permitted in any relevant BMJ Author's Self Archiving Policies, I confirm this Work has not been accepted for publication elsewhere, is not being considered for publication elsewhere and does not duplicate material already published. I confirm all authors consent to publication of this Work and authorise the granting of this licence.*

For peer review only

# "When you're hurt and you need serious help you call 999"

# Educating Children about Emergency Services and Appropriate use of 999: An Evaluation Study of the Blue Light Hub App


## Abstract

**Objectives.** In the face of unprecedented demand, the Welsh Ambulance Service developed 'Blue Light Hub': a new app to educate primary school-aged children about emergency services. Our overarching aim was to examine the effectiveness of the app.

**Design.** Primary school-aged children from three schools in South Wales, UK, played with the app for 2 hours over 2 weeks in class time. Children completed quizzes to assess their knowledge and awareness of, and confidence engaging with, emergency services before and after using the app.

**Participants:** Our evaluation focused on $N$ = 393 children who completed both the pre- and post-test quizzes. On average, children were 8-9 years (median school year, Year 4); 47.8% were male and 50.9% were female.

**Results.** After using the app, there was a significant increase in the proportion of children who knew of appropriate actions to take in non-emergency scenarios, $\chi^2(1) = 26.01$, and could provide a question a call handler would ask them if they called 999, $\chi^2(1) = 13.79$. There was also an increase in the proportion of children who could identify an NHS service that could help them if they were unwell, $\chi^2(1) = 33.31$, name different roles in the NHS, $\chi^2(1) = 12.80$, and knew how dialling 111 could help them $\chi^2(1) = 90.05$ (all $ps < .001$).

**Conclusion.** To our knowledge, Blue Light Hub is the first app of its kind designed to educate primary school-aged children about emergency services. Our findings provide preliminary evidence that the app supports children's knowledge and awareness of emergency services.




For peer review only
For peer review only - http://bmjopen.bmj.com/site/about/guidelines.xhtml

**Strengths and limitations of this study**

- A key strength of this study is the large sample of participants who engaged with the Blue Light Hub app in school time.
- Information regarding how teachers implemented the app with pupils (such as group size, level of teacher support) was not available.
- This app was developed during the social restrictions brought about by the Covid-19 pandemic; future apps should be co-produced with children, parents, and teachers.

For peer review only



For peer review only - http://bmjopen.bmj.com/site/about/guidelines.xhtml

## Introduction

The Welsh Ambulance Service handles more than 1800 calls a day from across the country. Yet of the 470,653 incidents recorded by the service in 2021, nearly a quarter were judged as non-essential and non-urgent [1]. Some reasons for non-essential engagement with emergency services may include misjudgement of emergency scenarios and perception of risk, a lack of awareness of other healthcare services, and a perception of convenience [2, 3, 4].

In the face of unprecedented demand on the service, a key priority of the Welsh Ambulance Service is to find avenues to maintain engagement with schools and influence the school curriculum to educate pupils about the Trust's services and ways to use health services appropriately [1]. Immersive and interactive experiences are an effective way to educate and familiarise children with NHS services and reduce anxieties about engaging with them, by giving children and young people opportunities to talk to NHS staff, handle equipment, and observe what might happen in different medical scenarios [5].

However, in-person interactive initiatives are resource and time-intensive and have limited opportunities for repeated exposure. Interactive apps are a useful and popular tool that is increasingly being integrated in classrooms [6]. Evidence suggests that interactive apps are a promising tool for supporting academic development [7] and for addressing public health challenges [8] such as health education [9]. By combining play with technology, interactive apps are low cost, easily distributed, and have the potential to actively engage children with material via feedback and rewards delivered through gameplay and repetition to reinforce knowledge [7, 10].

The Blue Light Hub App was designed by the Welsh Ambulance Service Trust to educate 7-to-11-year-olds about emergency services. The key objectives in the design of the interactive app were to teach children to recognise emergency from non-emergency scenarios and awareness of other services available in non-emergency situations (i.e., 111), about what happens when they call 999, the appropriate use of 999, how ambulance resources are dispatched and managed and the different uniforms they might encounter on their NHS journey. To the best of our knowledge, there is currently



For peer review only - http://bmjopen.bmj.com/site/about/guidelines.xhtml

no other interactive app that aims to support children's engagement with emergency services. In this evaluation, we examined the following questions: Does the use of the Blue Light Hub app increase children's (1) knowledge of (2) awareness about, and (3) confidence in using emergency services? These questions were investigated in the context of the Blue Light Hub App being used by primary school-aged children for two weeks in the classroom.

## Method

### Participants

Three primary schools in South Wales were invited to participate in the Blue Light Hub App evaluation. Ethical approval for the evaluation was obtained from the Cardiff University School of Psychology Ethics Committee (EC.21.12.14.6490R). For inclusivity, all children were invited to engage with the app and complete the quizzes. Consent was collected from caregivers for use of their child's data for the study. Our analyses focus on $N = 393$ children who completed both the pre- and post-test quizzes ($n = 115$ from School A, $n = 154$ from School B, and $n = 124$ from School C). On average, children were 8-9 years (median Year 4; IQR Year 3 [7-8 years] – Year 5 [9-10 years]), $n = 188$ (47.8%) were male, $n = 200$ (50.9%) were female (information on gender was missing for 5 participants). Sociodemographic information about the schools was obtained from their most recent school inspection (Estyn) reports. At School A, approximately 37% of pupils were eligible for free school meals in their 2017 report, and at Schools B and C nearly 10% and 8% of pupils were eligible for free school meals, according to their 2016 and 2019 reports respectively (rolling three-year national average in 2021 for pupils of statutory school age who are eligible for free school meals in primary schools in Wales is 21% [11]).

### Data Collection

The Blue Light Hub App is a fully bilingual (English-Welsh) gaming app designed by Welsh Ambulance Service NHS Trust and developed by DotC Studios [12]. The app comprises four games



For peer review only - http://bmjopen.bmj.com/site/about/guidelines.xhtml

(see Figure 1). After completing each game, children receive points that equate to money they can spend in the app's shop to buy accessories for their customisable avatar.

The Blue Light Hub App evaluation took place over 2 weeks in June 2022. Teachers were provided with written instructions for taking part in the evaluation. The instructions detailed that children were to be informed they could withdraw from the evaluation at any time and receive minimal support when completing their quizzes (support with reading and writing only). Children completed the first quiz in class time. After completion of the first quiz, teachers were asked to use the Blue Light Hub App with children in their class for 1 hour a week for the following 2 weeks. After these 2 weeks, children completed Quiz 2. In each quiz, children were asked questions designed to assess their knowledge about emergency scenarios, their awareness of different healthcare services and their confidence in accessing support from emergency services. Nine questions were duplicated from Quiz 1 (pre-test quiz) to Quiz 2 (post-test quiz) to enable the comparison of knowledge before and after using the Blue Light Hub App. All children received certificates from the Welsh Ambulance Service for taking part.

**Data Analysis.**

Quiz responses were analysed using SPSS software V.27 (IBM). Change in knowledge, awareness, and confidence before and after use of the Blue Light Hub app was assessed using McNemar's tests, to examine differences in proportions in children's responses from pre- to post-test quizzes. Inflated alpha levels due to multiple testing were addressed with a Bonferroni adjusted significance criterion of $p < .006$. A G*Power post hoc power analysis for McNemar's analysis indicated that a sample size of $N = 393$ yielded statistical power at .84 at an alpha level of .02 for proportion of discordant pairs .20, odds ratio = 2.

**Public and Patient Involvement Statement.**

There was no public or patient involvement in the study design, analysis, or interpretation of the results. However, pilot participants completed the Quiz items to ensure they were appropriate for the age range.



For peer review only - http://bmjopen.bmj.com/site/about/guidelines.xhtml

## Results

Table 1 shows quiz questions of interest and children's responses. For children's knowledge about emergency services, following use of the Blue Light Hub app a higher proportion of children responded that a patient should be taken to hospital in a car if they had a minor injury (hurt arm), $\chi^2(1) = 26.01$, $p < .0005$, $OR = 4.38$, 95% $CI$(2.47 to 7.77), and provided a correct question that a call handler would ask them if they called 999, $\chi^2(1) = 13.79$, $p < .0005$ ($OR = 28.48$, 95% $CI$(8.02 to 101.10); but it is worthy of note that a very high proportion of children correctly answered this question at both time points. There was no significant difference in the proportion of children who reported knowing what to do if a someone had stopped breathing, $p = .18$. Children's follow-on free text responses to "If yes, what would you do?" were coded for correct actions (e.g., calling 999, administering cardiopulmonary resuscitation [CPR]). Similarly, there was no significant increase in children who said they knew how to help and gave a correct action, $p = .07$. The increase in the proportion of children who could correctly describe an emergency was also not significant, $p = .06$.

**Table 1. Children's quiz results before and after playing with the Blue Light Hub app**

| | Quiz 1 (Pre-test) | Quiz 2 (Post-test) |
|---|---|---|
| Knowledge of Emergency Services | | |
| "If someone you know hurt their arm and they needed to go to hospital, what would you do?" *n* (%) take in car vs. call 999 | **70/360 (17.8)** | **126/374 (32.1)** |
| "If you saw someone and they had stopped breathing, would you know what to do?" *n* (%) yes | 306/390 (78.5) | 319/392 (81.4) |
| "If yes, what would you do?" *n* (%) correct response | 275/306 (89.9) | 299/319 (93.7) |
| "When you ring 999 for an ambulance, we ask lots of questions. Can you think of 1 question we ask you?" *n* (%) correct response | **360/393 (91.6)** | **381/393 (96.9)** |

For peer review only



For peer review only - http://bmjopen.bmj.com/site/about/guidelines.xhtml

| | | |
|---|---|---|
| You should only phone 999 for an ambulance in an 'emergency'. What do you think an 'emergency' is?" *n* (%) correct response | 351/393 (89.3) | 366/393 (93.1) |
| Awareness of Healthcare Services | | |
| "If you are not feeling very well, your family and teachers can help you. Can you think of anyone else who can help you if you are not feeling very well?" *n* (%) reporting NHS service | **235/393 (58.8)** | **294/393 (74.8)** |
| "There are lots of people that do different jobs in the Ambulance Service. Can you name 2 of them?" *n* (%) correct responses | **229/393 (58.3)** | **270/393 (68.7)** |
| "Do you know how 111 can help you?" *n* (%) yes | **101/391 (25.7)** | **226/393 (57.5)** |
| Confidence Calling 999 | | |
| "Would you feel confident if you had to ring 999 for an ambulance?" *n* (%) yes | 289/389 (73.5) | 298/386 (75.8) |

*Note.* Bold represents significant differences in proportions from the pre- to post-test quizzes.

In terms of children's awareness of healthcare services, we found that following use of the Blue Light Hub app, there were significant increases in the proportion of children who identified an NHS service that could help them if they were not feeling well, $\chi^2(1) = 33.31$, $p < .0005$, $OR = 9.94$, 95% $CI$(5.76 to 17.15), in children who could name two roles in the NHS, $\chi^2(1) = 12.80$, $p < .0005$, $OR = 4.35$, 95% $CI$(2.76 to 6.84), and in children reporting that they knew how dialling 111 could help them, $\chi^2(1) = 90.05$, $p < .0005$, $OR = 3.54$, 95% $CI$(2.09 to 5.99), (see Table 1).

Finally, we did not identify a significant change in the proportion of children who reported feeling confident if they had to ring 999 for an ambulance $p = .31$.



For peer review only - http://bmjopen.bmj.com/site/about/guidelines.xhtml

## Discussion

To our knowledge, the Blue Light Hub App designed by the Welsh Ambulance Service Trust is the first of its kind to teach primary school-aged children about emergency services. Our findings suggest that using the Blue Light Hub app for just two hours over two weeks during school time was related to improvements in children's knowledge about, and awareness of, emergency services.

Importantly, following use of the app children demonstrated better understanding of when *not* to call an ambulance, suggesting the app offers opportunities to better understand actions to take in non-emergency medical scenarios. We also found that children were more likely to list NHS services that could help them if they were feeling unwell, they demonstrated a better awareness of different NHS job roles, and an increased awareness of how 111 could help them in non-emergency situations. Although we did not find significant increases in children's confidence if they were to call 999, it is noteworthy that three-quarters of children reported feeling confident and there was an increase in knowledge about what a call handler may ask them if they had to do so. Our findings extend previous research demonstrating that secondary school children tend to have high levels of knowledge about contacting emergency services [13, 14]; our evaluation indicates this is also true for younger primary school aged children. Our findings are particularly encouraging given that barriers to intervening in emergency scenarios later in childhood often involve lack of confidence, fear of making mistakes, and lack of awareness and knowledge of appropriate actions [15].

One of the main aims of developing the Blue Light Hub app was to find novel ways to support education about urgent and emergency care to address the rise in calls for ambulance services in non-emergency scenarios. Such calls add significant pressure to an already stretched service that needs to reduce spending [16]. Research shows that reasons for ambulance use in non-emergency situations are multifaceted [2, 3, 4]. For example, recent reviews have indicated numerous factors associated with ambulance use for non-emergency problems. These included socioeconomic status, geographical location, practical reasons (i.e., lack of transport), emotional reasons (i.e., uncertainty, reassurance), and level of education, knowledge, and experience in judging the appropriateness of ambulance use for their circumstances [17, 18]. Although a small-scale intervention may not radically



For peer review only - http://bmjopen.bmj.com/site/about/guidelines.xhtml

change the behaviour of the population, better knowledge and awareness repeatedly consolidated by use of educational apps may encourage individuals to evaluate their options more thoroughly before seeking emergency care. Quite possibly, interventions such as the Blue Light Hub app may have most impact in more deprived areas; future research would do well to investigate this possibility on a larger scale and explore the generalisability of this type of education-based intervention in other locations and contexts. Although this investigation focused on improving awareness, knowledge, and confidence in primary school aged children, app-based education could also be explored as an avenue to supporting adult education about emergency services and decision making prior to calling an ambulance.

**Improvements**

The app was developed during the Covid-19 pandemic, and therefore opportunities for consultation with young people, parents, and teachers were limited. We recommend that future development of such apps involve the inclusion of both a young person and parent and teacher advisory groups alongside the lessons learned from this this initial evaluation.

In general feedback, children and teachers shared that the Blue Light Hub app was engaging and enjoyable to use. However, this initial evaluation gave insights into ways the app can be improved in terms of accessibility and improving children's learning experiences. Some teachers suggested that the app should include audio to reduce the need for reading skills and increase accessibility of the app. Another popular suggestion by teachers was further consideration that children typically share tablet devices in the classroom and across year groups. An important step for further development of the app would be to ensure that children can access their own profiles to be able to keep track of their own progress and learning journey.

Children had many ideas for more games they would want to play on the app, including how to give CPR, first aid, and more immersive experiences of being an ambulance or air ambulance driver. Indeed, one valuable direction for game development could be an experience of steps to take if



For peer review only - http://bmjopen.bmj.com/site/about/guidelines.xhtml

a child encountered a patient who required CPR, given that evidence suggests that in later childhood, despite good knowledge of actions to take, this does not necessarily translate to practical ability [19].

**Limitations**

Although these findings are promising, there are limitations to note. This evaluation represents a first step in understanding the feasibility and effectiveness of an educational app to support children's knowledge of emergency services, however, future evaluation of the effectiveness of app as an intervention for children should be investigated in the context of a randomized controlled trial to evaluate the effectiveness of the app in the short- and long-term, that also accounts for the level of support that teachers provide when navigating the app. Given the short-term use of the app in this evaluation, a further avenue for investigation will be the long-term retention of knowledge and the potential need for repeated use of the app in class time. Additionally, future research would do well to understand how the app can be used to consolidate children's knowledge of emergency services alongside in-person interactive initiatives.

**Conclusion**

In summary, our findings suggest that primary school aged children's knowledge and awareness of emergency scenarios and services can be supported via educational apps, such as the Blue Light Hub app. Our findings corroborate research demonstrating the value of educating children in what to do in emergency scenarios and emergency care [5], and that primary school-aged children are receptive to interventions that support their ability to recognise emergencies and call for help [20, 21]. We join other calls for more emphasis on emergency response readiness in school curriculums [15, 22, 23]; this may have positive consequences for wider community knowledge of appropriate use of emergency services. Our findings, though preliminary, suggest that amongst other educational approaches, educational apps may be a useful resource for teaching.

For peer review only



For peer review only - http://bmjopen.bmj.com/site/about/guidelines.xhtml

**Contributorship statement.** Author AP designed the study, monitored data collection, planned the analyses, drafted, and revised the paper. Author FM led recruitment, monitored data collection, and edited the paper. We would also like to thank Cara Houlcroft, Meg Kydd-Coutts, Maisie Bowmaster, and Anna Preece for their research assistance. We would like to thank the children and teachers who took part in this study. PPI Statement: Patients or the public were not involved in the design, or conduct, or reporting, or dissemination plans of our research.

**Competing interests:** This research was conducted in consultation with the Welsh Ambulance Service NHS Trust (WAST), but WAST did not have final say in the study design nor played a direct role in the collection or analysis of the data; interpretation of the results; or writing of the manuscript.

**Funding:** Dr. Amy L. Paine is funded by an ESRC New Investigator Award (Grant reference: ES/T00049X/1).

**Data sharing statement:** Data are available from the research team upon reasonable request.

For peer review only



For peer review only - http://bmjopen.bmj.com/site/about/guidelines.xhtml

## References

1. Welsh Ambulance Service NHS Trust. '999? I've eaten a mouldy tomato!' – Inappropriate calls to Welsh Ambulance Service revealed [online]. 2022. https://www.ambulance.wales.nhs.uk/Default.aspx?gcid=1862&lan=en#:~:text=THE%20Welsh%20Ambulance%20Service%20has%20revealed%20the%20most,Department%2C%20while%20another%20dialled%20999%20for%20a%20papercut (accessed 21 March 2023).

2. Ahl C, Nyström M, Jansson L. Making up one's mind: – Patients' experiences of calling an ambulance. Accident and Emergency Nursing 2006; 14: 11-19. doi: 10.1016/j.aaen.2005.10.002

3. Brooker MJ, Shaw ARG, Purdy S. Why do patients with 'primary care sensitive' problems access ambulance services? A systematic mapping review of the literature. BMJ Open 2015; 5: e007726. doi: 10.1136/bmjopen-2015-007726

4. Chapman B, Turnbull T. Health professionals' perceptions of inappropriate use of A&E services. British Journal of Nursing 2016; 25. doi: 10.12968/bjon.2016.25.9.476

5. Allen D. 999 Club aims to reduce children's anxieties about emergency care. Emergency Nurse 2018; 25: 8. doi: 10.7748/en.25.10.8.s8

6. Haßler B, Major L, Hennessy S. Tablet use in schools: a critical review of the evidence for learning outcomes. Journal of Computer Assisted Learning 2016; 32: 139–156. doi: 10.1111/jcal.12123

7. Griffith SF et al. Apps As Learning Tools: A Systematic Review. Pediatrics 2020; 145: e20191579. 10.1542/peds.2019-1579

8. World Health Organization. WHO Guideline: Recommendations on Digital Interventions for Health System Strengthening [online]. 2019. https://apps.who.int/iris/bitstream/handle/10665/324998/WHO-RHR-19.7-eng.pdf (accessed 21 March 2023).



For peer review only - http://bmjopen.bmj.com/site/about/guidelines.xhtml

9. Brown JM et al. Optimizing Child Nutrition Education with the Foodbot Factory Mobile Health App: Formative Evaluation and Analysis. JMIR Formative Research 2020; 4:e15534. doi: 10.2196/15534

10. Hirsh-Pasek K et al. Putting education in "educational" apps: lessons from the science of learning. Psychological Science in the Public Interest, 2015; 16: 3–34. doi: 10.1177/1529100615569721

11. Estyn. The teaching of Welsh history including Black, Asian and Minority Ethnic history, identity and culture [online] 2021. https://www.estyn.gov.wales/document-page/20673/appendices/appendix-3#:~:text=The%20rolling%20three-year%20national%20average%20in%202021%20for,Wales%20have%20a%20special%20educational%20needs%20provision%20%28SEN%29 (accessed 21 March 2023).

12. DotC Studios [online]. 2023. https://dotcstudios.com/ (accessed 21 March 2023).

13. Kanstad B K et al. CPR knowledge and attitude to performing bystander CPR among secondary school students in Norway. Resuscitation, 2011, 82: 1053-1059. doi: 10.1016/j.resuscitation.2011.03.033

14. Schuffelen P. Knowledge of the European emergency number in secondary schools for different grades and education level groups. Resuscitation, 2013; 84(Suppl 1): S40. doi: 10.1016/j.resuscitation.2013.08.105

15. Parnell M M et al. Knowledge of and attitudes towards resuscitation in New Zealand high-school students. Emergency Medicine Journal, 2006, 23: 899-902. doi: 10.1136/emj.2006.041160

16. NHS England. High quality care for all, now and for future generations: Transforming urgent and emergency care services in England. The evidence base from the urgent and primary care review [online] 2013. https://www.england.nhs.uk/wp-content/uploads/2013/06/urg-emerg-care-ev-bse.pdf

17. Proctor A et al. What factors are associated with ambulance use for non-emergency problems in children? A systematic mapping review and qualitative synthesis. BMJ Open, 2021, 11(9): e049443. doi: 10.1136/bmjopen-2021-049443



For peer review only - http://bmjopen.bmj.com/site/about/guidelines.xhtml

18. Booker M J et al. Seeking ambulance treatment for 'primary care' problems: a qualitative systematic review of patient, carer and professional perspectives. BMJ Open, 2017, 7(8): e016832. doi: 10.1136/bmjopen-2017-016832

19. Lester C et al. Teaching schoolchildren cardiopulmonary resuscitation. Resuscitation, 1996, 31(1): 33-88. doi: 10.1016/0300-9572(95)00912-4

20. Bernardo L M et al. Basic Emergency Lifesaving Skills (BELS): A framework for teaching skills to children and adolescents. International Journal of Trauma Nursing, 2002, 8: 48-50. doi: 10.1067/mtn.2002.123027

21. Wilks J et al. Emergency response readiness for primary school children. Australian Health Review, 2015; 40: 357-363. doi: 10.1071/AH15072

22. Bollig G et al. Primary school children are able to perform basic life-saving first aid measures. Resuscitation, 2009; 80: 689-692. doi: 10.1016/j.resuscitation.2009.03.012

23. Uray T et al. Feasibility of life-supporting first-aid (LSFA) training as a mandatory subject in primary schools. Resuscitation, 2003; 59: 211-220. doi: 10.1016/S0300-9572(03)00233-8

For peer review only

Figure caption:

**Figure 1. The Blue Light Hub App**

For peer review only



For peer review only - http://bmjopen.bmj.com/site/about/guidelines.xhtml

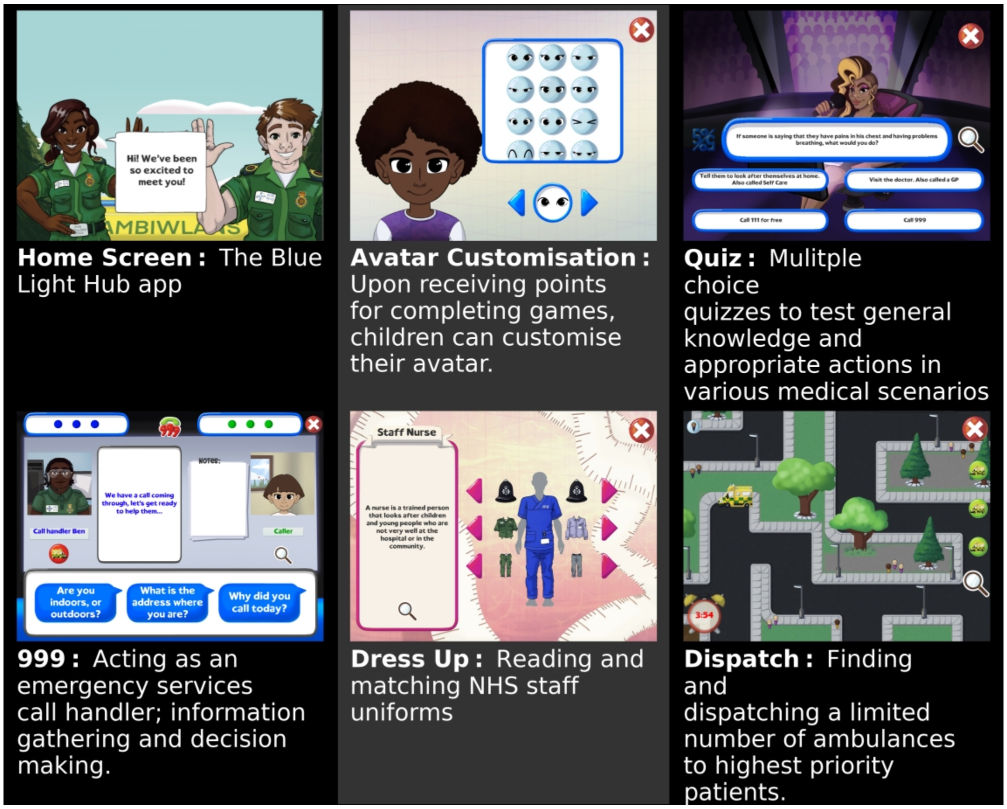


Figure 1. The Blue Light Hub App

149x119mm (600 x 600 DPI)
